# An Investigation on the Risk Factors of Thyroid Diseases in Community Population in Hainan

**DOI:** 10.1155/2022/4514538

**Published:** 2022-07-09

**Authors:** Tuanyu Fang, Leweihua Lin, Qianying Ou, Lu Lin, Huachuan Zhang, Kaining Chen, Huibiao Quan, Yangli He

**Affiliations:** ^1^Department of Endocrinology, Hainan General Hospital, Hainan Affiliated Hospital of Hainan Medical University, Haikou, China; ^2^Department of Health Care Centre, Hainan General Hospital, Hainan Affiliated Hospital of Hainan Medical University, Haikou, China

## Abstract

**Background:**

In recent years, the incidence of thyroid diseases has increased significantly, which has seriously affected people's work and life. The purpose of this study was to explore the epidemiological characteristics of thyroid diseases and autoantibodies.

**Method:**

According to the principle of overall sampling, resident residents ≥18 years and who will not move within 5 years are randomly selected. A total of 2136 eligible individuals were divided into case and control groups according to whether they have thyroid disease. Finally, the impact of potential risk factors on thyroid diseases was evaluated.

**Results:**

The overall prevalence of thyroid disease was 58.3%, and there was a significant difference in the prevalence of thyroid disease between women and men (*p* = 0.004). Except for the age group ≥70 years, with the increase in age, the prevalence gradually increased (*p* < 0.05). Participants with positive thyroid autoantibodies (TPOAb or TgAb) had a higher prevalence than participants with negative autoantibodies. The positive rate of autoantibodies in women was higher than that in men (*p* < 0.05). UIC (*p* = 0.004) and free thyroid hormone (FT4) (*p* = 0.001) levels of men were higher than those of women, and the TSH level of women was higher than that of men (*p* = 0.002). The regression analysis showed that women, older age, and family history of thyroid disease were independent risk factors for thyroid disease.

**Conclusion:**

The prevalence of thyroid diseases in Hainan was high. Women are more susceptible to thyroid disease than men, and the prevalence increased with age.

## 1. Introduction

With the intensification of environmental endocrine disruptors, the acceleration of work rhythm, and changes in diet, the incidence of thyroid diseases has a clear upward trend, which has seriously affected people's work and life. Regarding the prevalence of thyroid disease in the population, the landmark British Whickham study found that 7.5% of women and 2.8% of men over the age of 20 years have TSH>6 m U/L. They also found that the prevalence of subclinical hypothyroidism and clinical hypothyroidism was 5% and 0.1%, respectively, and the prevalence rate of hyperthyroidism was 1.6% [1]. However, in subsequent studies, the prevalence of thyroid diseases in the Netherlands [2], Spain [3], the United States [4], Australia [5], and Japan [6] was not the same. It can be seen that there were differences in the prevalence of thyroid dysfunction in different regions and different populations.

There were many factors that affect thyroid function. In addition to regional and ethnic factors, it is also related to factors such as gender, age, and thyroid autoantibodies. It is generally believed that the prevalence of abnormal thyroid function in women was higher than that in men [1–9]. In terms of TSH alone, many studies have reported that the average serum TSH level of women was higher than that of men of the same age [4, 10]. It is generally believed that TSH is the main hormone that stimulates polyiodine and regulates thyroid function [11]. Regarding the influence of age on thyroid function, it is currently believed that with the increase in age, the prevalence of hypothyroidism gradually increases [3–8]. Positive thyroid autoantibody was a risk factor for abnormal thyroid function, which has almost become a recognized fact in the academic circles [2–4, 6, 12, 13]. Scholars, such as Eskelinen, believe that TPOAb and TgAb positive did not affect the normal range of FT4 but only increased the upper limit of the women TSH range. They believed that the positive thyroid autoantibody had less significant impact on thyroid function than imagined [14]. It can be seen that autoantibodies are closely related to thyroid function. However, which antibody has a more significant impact on which indicator of thyroid function is still controversial [13].

The purpose of our study is to determine the possible factors affecting the prevalence of thyroid diseases and the epidemiological characteristics of thyroid diseases in Hainan. This study will provide valuable references for the prevention, diagnosis, and treatment of thyroid diseases.

## 2. Materials and Methods

### 2.1. Survey Subject

From September 2019 to September 2020, a stratified cluster sampling was conducted to select resident residents aged ≥18 years in Hainan area who will not move within 5 years. The inclusion criteria of the survey subjects are as follows: [1] age ≥18 years; [2] those who have lived at the survey site for more than 5 years; [3] those who have not received an iodine-containing contrast agent examination or have taken amiodarone within the past three months; and [4] exclude women who are already pregnant. This research was approved by the ethics committee of Hainan. Finally, 2136 participants who met the above conditions were selected as the survey subjects.

### 2.2. Method

All the staff participating in this research will take up their posts after being uniformly trained and qualified.

#### 2.2.1. Laboratory Sample Collection and Measurement

In this study, thyroid testing, thyroid function testing, urine iodine testing, and questionnaire surveys were performed on all subjects. (1) Thyroid examination: we used a color Doppler ultrasound diagnostic apparatus (GE, LOGIQ*α*100, USA) for thyroid examination and set the probe frequency to 7.5 MHz. After the examination, we calculated the thyroid volume according to the WHO formula. (2) Thyroid function test: we collected 10 ml of fasting venous blood from participants. The samples were left at room temperature (20–25°C) for 2-3 hours, then centrifuged (3000 r/min, 10 minutes) to separate the serum. Finally, they were stored at −20°C. A solid-phase chemiluminescence enzyme-immunoassay method was used to detect the levels of thyroid stimulating hormone (TSH), thyroid peroxidase antibody (TPOAb), and thyroglobulin antibody (TgAb). For people whose TSH was outside the normal range, free thyroid hormone (FT4) and free triiodothyronine (FT3) were additionally tested. (3) Urine specimen sampling: we took 5 ml of fasting urine; arsenic and cerium catalysis spectrophotometric methods were used to detect urinary iodine concentration (UIC) [15].

#### 2.2.2. Diagnostic Criteria

The reference ranges of thyroid function indexes TSH, FT4, and FT3 are 0.27∼4.2 mIU/L, 12.0∼22.0 pmol/L, and 3.1∼6.8 pmol/L, respectively. TPOAb positive ≥35 IU/L; TgAb positive ≥115 IU/L. The functional sensitivity of serum TSH determination was 0.014 lIU/mL. The intraassay coefficient of variation (CV) of serum TSH, FT4, FT3, TPOAb, TgAb, and TRAb was 1.1–6.3%, and the interassay CV was 1.9–9.5%, which ensured the reproducibility of serum TSH, FT4, FT3, TPOAb, and TgAb determinations. FT4, FT3, and TSH values were all lower than the normal range was hypothyroidism. FT4 and FT3 were normal but the TSH value was lower than the normal range was subclinical hypothyroidism. FT4, FT3, and TSH values were higher than the normal range was hyperthyroidism. FT4 and FT3 were normal but the TSH value is higher than the normal range was subclinical hyperthyroidism. The standard materials of the Chinese Center for Disease Control and Prevention (GBW09108, GBW9109, and GBW9110) were used as controls to control the quality of urine iodine determination experiments. According to the urine iodine level, the participants were divided into the following 4 groups: iodine-deficiency group (<150 *μ*g/L), iodine-sufficient group (150∼249 *μ*g/L), iodine-overdosing group (250∼499 *μ*g/L), and iodine-overdosing group (≥500 *μ*g/L) [16].

#### 2.2.3. Statistical Analysis

SPSS 24.0 was used for analysis. Counting data were expressed in frequency and percentage. The morbidity rate was compared using the *χ*^2^ test. Measurement data were expressed as “mean ± standard deviation” (mean ± SD). TSH, UIC, TPOAb, and TgAb are skewed and expressed by the median (25–75 percentile). Statistical methods included *t* test, *χ*^2^ test, Mann–Whitney *U* test, and so on. Finally, logistic regression analysis was used to evaluate independent risk factors of thyroid disease epidemic. *p* < 0.05 was considered statistically different.

## 3. Result

### 3.1. Survey of Research Objects

After the questionnaire survey and physical examination, a total of 2136 participants participated in the study after excluding those who did not retain serum and urine specimens and had incomplete data. The average age was 47.49 ± 13.29 years (18–86 years). The basic information obtained through physical examination or questionnaire survey is shown in Table 1.

### 3.2. Prevalence of Thyroid Diseases in Different Genders

Among all participants, 1,245 patients were with thyroid disease. The prevalence of thyroid disease in women is higher than that in men, which was 73.6% and 26.4%, respectively. As shown in Table 2, the prevalence of various thyroid diseases was significantly different in gender (*p* = 0.004). Except for hyperthyroidism and thyroid tumors, the prevalence of other thyroid diseases differs greatly between men and women.

### 3.3. Gender Differences in the Prevalence of Thyroid Diseases in Different Age Groups

**Figure 1:**
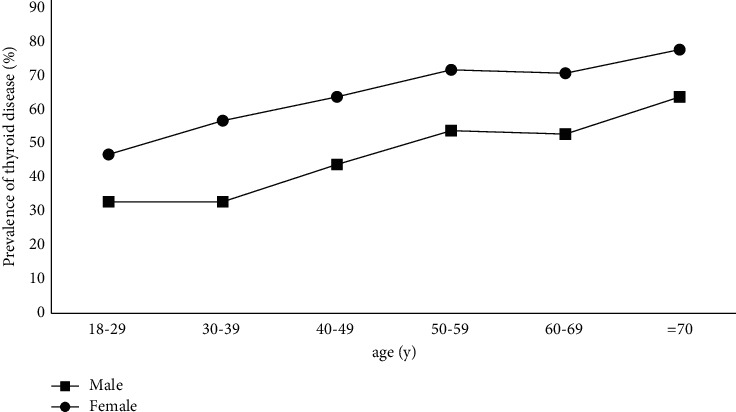
Prevalence of thyroid diseases in different age groups.

Within the scope of this study, the overall prevalence of thyroid disease was 58.3% (1245/2136). Our survey results showed (Table 3) that, excluding the age group ≥70 years, the prevalence of thyroid disease increases with age (Figure 1). The prevalence of thyroid disease in women among all age groups was significantly higher than that in men (18–29: *p* = 0.046; 30–39: *p* < 0.001; 40–49: *p* < 0.001; 50–59: *p* < 0.001; and 60–69: *p* = 0.002).

### 3.4. Differences in Prevalence Rates of Thyroid Diseases for Different Obesity Parameters

#### 3.4.1. BMI

We divided the subjects into normal (18.5 ≤ BMI<24), underweight (<18.5), overweight (24 ≤ BMI<28), and obese (≥28) according to BMI. Overall, there were significant differences in the prevalence of thyroid disease between obese and normal subjects (*p* = 0.006; Table 4). The prevalence of thyroid disease in normal subjects is higher than that in underweight, overweight, or obese subjects. In addition, there is no significant difference in the prevalence of thyroid disease between underweight, overweight, and normal people. In addition, there is no significant difference in the prevalence of thyroid diseases between normal subjects and underweight or overweight subjects.

#### 3.4.2. Waist Circumference (WC)

We divided the subjects into central obesity and noncentral obesity according to WC. Central obesity is defined as waist circumference ≥90 cm in men or 85 cm in women, otherwise it is noncentral obesity. Overall, there was a significant difference in the prevalence of thyroid disease between central obesity and noncentral obesity subjects (*p* < 0.001; Table 4), and the prevalence of thyroid disease in the central obesity group was lower than that in the noncentral obesity group.

### 3.5. Differences in the Prevalence between Autoantibody Positive and Negative Groups

**Figure 2:**
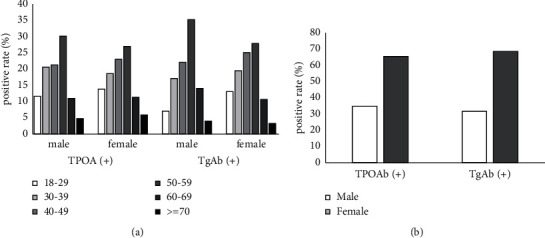
(a) Positive rates of autoantibodies in different age groups. (b) Positive rates of autoantibodies in different gender groups.

Overall, participants with positive thyroid autoantibodies (TPOAb positive or TgAb positive) had a higher prevalence of thyroid disease than negative participants (Table 5). The results of the Mann–Whitney *U* test showed (Table 5) that this difference was statistically significant (*p* < 0.001). In addition, we also investigated the differences in the positive rate of autoantibodies among patients with thyroid disease in different age and gender groups. The results showed that the positive rate of autoantibodies in different age groups was not significantly different in gender (Table 6 and Figure 2(a)). However, the positive rate of autoantibodies in women is higher than that in men (TPOAb: 65.3% Vs. 34.7%; TgAb: 68.4% Vs. 31.6%) (Table 6, Figure 2(b)).

### 3.6. Gender Differences in Different Thyroid Hormone Levels

Our survey results showed (Table 7) that female TSH levels were significantly higher than that in males (median: 1.88 Vs. 1.67, *p* = 0.001). Women's UIC (median: 147.30 Vs. 159.32, *p* = 0.004) and FT4 (median: 15.5 Vs. 17.14, *p* = 0.002) levels were significantly lower than men's. There was no significant difference in FT3 levels between male and female groups.

### 3.7. The Relationship between the Prevalence of Thyroid Diseases and the Level of UIC

**Figure 3:**
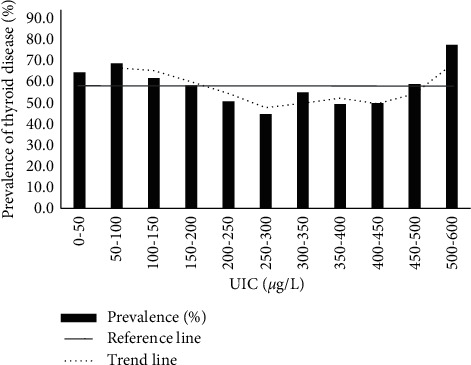
Prevalence of thyroid diseases in different UIC levels.

We divided the subjects into 11 groups according to UIC level from high to low (0 *μ*g/L∼, 50 *μ*g/L∼, 100 *μ*g/L∼, 150 *μ*g/L∼, 200 *μ*g/L∼, 250 *μ*g/L∼, 300 *μ*g/L∼, 350 *μ*g/L∼, 400 *μ*g/L∼, 450 *μ*g/L∼, and 500 *μ*g/L∼). As shown in Figure 3, when 150 *μ*g/L∼200 *μ*g/L < UIC > 300 *μ*g/L, the prevalence of thyroid diseases decreased significantly; when UIC >350 *μ*g/L, the prevalence showed an upward trend (Table 8). Especially when UIC >500 *μ*g/L, the prevalence increased significantly. In general, the prevalence of thyroid disease and UIC level of the participants in this study showed a U-shaped curve (Figure 3).

### 3.8. Independent Risk Factor Assessment

We used gender, age, BMI, smoking/drinking status, region, season, family history of thyroid disease, hyperlipidemia, hyperuricemia, hypertension, and other factors as a model to evaluate potential risk factors related to the occurrence of thyroid disease. The results showed (Table 9) that female gender was a risk factor for thyroid disease; participants aged 40–69 years have a significantly higher risk of thyroid disease compared with participants aged 18–29 years (40∼49 years: *p* < 0.001; 50∼59 years: *p* = 0.009; 30∼69 years: *p* = 0.037). Among them, the OR value of the 60∼69-year group was 5.2 times that of the 18∼29-year group. Participants who had no family history of thyroid disease had a reduced risk of thyroid disease. We did not find that other factors were independent risk factors for thyroid disease.

## 4. Discussion

Thyroid diseases are frequent and common diseases in endocrine system. In recent years, with the improvement of people's living standards, increased awareness of health care, and great changes in dietary results, the incidence of thyroid diseases has also shown an upward trend [17].

The survey results showed that 58.3% of the Hainan community population suffers from thyroid disease, of which 73.6% are females and 26.4% are males. This result was similar to the results of an epidemiological study in a Mediterranean population: Lucas et al. found that women with abnormal thyroid function accounted for 71.15% [3]. The above study showed that there were obvious gender differences in the prevalence of thyroid diseases in the Hainan population. Fan et al. found that the prevalence of thyroid nodules (TN) in women in Tianjin, China, was significantly higher than that in men [18]. A survey by Zimmermann and Boelaert found that the prevalence of TN in men was lower than that in women [19]. The fact that women are more likely to suffer from TN than men has also been found in many areas [20–22]. However, our results indicated that men (70.8%) had a higher prevalence of thyroid nodules than women (58.7%). We speculate that the reason for this difference may be caused by differences in the genetic background, living habits, and iodine intake of the research subjects. Further investigation and research is needed to verify our results.

In this study, regardless of men and women, the prevalence of thyroid disease was increasing with age. Except for the age group ≥70 years, the prevalence of thyroid disease in women in all age groups was significantly higher than that in men. Camargo et al. conducted an epidemiological survey of thyroid diseases in São Paulo, Brazil, and found that the prevalence of women increased with age [9]. Especially in the two age groups 40∼49 and 50∼59 years of age, the gender difference in thyroid prevalence was particularly obvious. It is well known that women of these two age groups are close to menopause or already have menopause, and their hormone levels are prone to be unstable. In addition, people with MetS (metabolic syndrome) have a higher probability of abnormal thyroid function [23, 24], and MetS is related to sex hormones [25, 26]. In summary, we speculate that sex hormones may be related to the occurrence of thyroid disease.

A number of studies have confirmed that obesity is a risk factor for thyroid diseases, such as hypothyroidism and thyroid cancer [27–29]. However, our results seemed to be slightly different from previous studies. The investigation results showed whether subjects were divided according to body mass index or waist circumference; the prevalence of thyroid disease of subjects defined as obesity is lower than that of normal subjects. The above fact suggests that obesity does not appear to be a risk factor for thyroid disease within the scope of our investigation. We speculated that the differences may be caused by the differences in the investigated region, genetic background, or body weight distribution between our study and previous studies. However, this is only a guess. It is necessary to further expand the sample size to verify the association between obesity and thyroid disease risk.

In this study, the prevalence of thyroid disease among autoantibody-positive participants was higher than that of autoantibody-negative participants. We did not find that the positive rate of autoantibodies was related to the age of participants, which is consistent with the results of previous studies [5, 12]. However, both NHANES III in the United States and Nijmegen in the Netherlands have found strong evidence that the positive rate of autoantibodies was associated with age [2, 4]. We speculate that the reason for this difference may be the differences in age distribution, genetic background, etc. of the survey subjects in each study. In addition, the positive rate of autoantibodies in women was significantly higher than that in men (TPOAb: 65.3% Vs. 34.7%; TgAb: 68.4% Vs. 31.6%). This is consistent with the research results of the National Health and Nutrition Examination Survey (NHANES III) in the United States [4], The Health Study of Nord-Trondelag in Norway [12], Busselton in Australia [5], Catalonia in Spain [3], and Nijmegen in the Netherlands [2]. It can be seen that women who have a higher positive rate of autoantibodies will be more likely to suffer from thyroid disease than men. The correlation between the positive rate of autoantibodies and the age of participants still needs further investigation.

The results of this investigation showed that within the scope of our investigation, lower or higher UIC levels will increase the prevalence of thyroid, presenting a U-shaped curve. Our results were consistent with previous studies [30, 31]. Krohn et al. found that iodine deficiency was related to the molecular mechanism of thyroid nodules [32]. Thyroid nodules are most commonly found thyroid disease [33]. In this study, thyroid nodules have the highest prevalence of all thyroid diseases. In this study, iodine deficiency increased the prevalence of thyroid diseases, which may be because iodine deficiency promotes the occurrence of thyroid nodules. Excessive iodine can stimulate the response of thyroid lymphocytes and cause autoimmune thyroiditis [34]. In this survey population, the prevalence of iodine-excess thyroid disease will increase accordingly, which may be related to the occurrence of autoimmune thyroiditis easily caused by excess iodine. In this study, women's UIC levels were significantly lower than men's, so we suspect that women's higher prevalence of thyroid disease than men may also be related to female iodine deficiency.

In addition to finding gender differences in UIC levels in this study, we also found that men's TSH levels were significantly higher than women's. TSH is the main hormone that stimulates polyiodine and regulates thyroid function. Therefore, we speculate that the prevalence of thyroid in women is higher than that in men, which may also be related to the gender difference in TSH levels. In addition, this study found that gender, age, and family history of thyroid can be used as independent risk factors for thyroid disease in the logistic regression analysis. Previous studies have also reported other independent risk factors: hypertension [35], smoking [36], BMI [37], etc. However, these risk factors were not independent risk factors for thyroid disease in the participants in this study.

In summary, the prevalence of thyroid diseases, the positive rate of thyroid autoantibodies, the level of TSH, and their respective gender and age distribution characteristics are not the same in different studies. This may be caused by the selected population's regional selection [2, 4, 9, 38], ethnic inheritance, sample size [3–5, 7], age-sex ratio of research subjects [39, 40], iodine intake level [40], and the establishment of different detection methods and diagnostic criteria [40]. However, this study and many of the abovementioned research results showed that the prevalence of thyroid dysfunction was highly correlated with gender, age, and positive thyroid autoantibodies.

However, it is worth noting that our study has certain limitations. A large sample size and expansion of the survey area are necessary, which will help increase the reliability of the research results. In addition, we did not take into account other factors that could lead to thyroid diseases. Subsequent investigations should be conducted on potential risk factors for each thyroid disease, which is a better way to assess the risk factors for a particular thyroid disease.

## 5. Conclusion

The prevalence of thyroid diseases in Hainan women is higher than that in men, the prevalence of thyroid diseases increases with age, and there are gender differences in the levels of TSH, UIC, and FT4. Older age, female, and family history of thyroid are independent risk factors for thyroid disease in Hainan, China.

## Figures and Tables

**Table 1:** Characteristics of subjects.

Item	Thyroid cases (*n* = 1245)	Normal (*n* = 891)
Gender		
Male	329 (26.4%)	380 (42.6%)
Female	916 (73.6%)	511 (57.4%)
Age		
Mean ± SD	49.55 ± 0.38	44.86 ± 0.46
18–29	107 (8.6%)	141 (15.8%)
30–39	193 (15.5%)	201 (22.6%)
40–49	270 (21.7%)	199 (22.3%)
50–59	415 (33.3%)	216 (24.2%)
60–69	192 (15.4%)	108 (12.1%)
≥70	68 (5.5%)	26 (2.9%)
BMI		
BMI < 18.5	66 (5.3%)	50 (5.6%)
18.5 ≤ BMI < 24	616 (49.5%)	469 (52.6%)
24 ≤ BMI < 28	425 (34.1%)	289 (32.4%)
BMI ≥ 28	138 (11.1%)	83 (9.3%)
WC (cm)		
Male ≥ 95 and female ≥ 90	631 (50.7%)	455 (51.1%)
85 ≤ male < 95 and 80 ≤ female < 90	543 (43.6%)	377 (42.3%)
Male < 85 and female < 80	71 (5.7%)	59 (6.6%)
WHtR	0.51 ± 0.06	0.50 ± 0.06
Height	158.23 ± 0.23	160.39 ± 0.28
Weight	59.73 ± 0.32	60.75 ± 0.40
Systolic blood pressure	125.04 ± 0.59	123.15 ± 0.65
Diastolic blood pressure	78.23 ± 0.36	77.75 ± 0.39
Heart rate	81.89 ± 0.37	80.98 ± 0.39
TSH	2.85 ± 0.17	1.92 ± 0.03
Serum vitamin D3	37.12 ± 0.32	38.42 ± 0.44
Triglycerides	1.83 ± 0.05	1.92 ± 0.07
Total cholesterol	5.43 ± 0.03	5.34 ± 0.04
LDL		
Mean ± SD	2.95 ± 0.02	2.87 ± 0.03
Low	11 (0.9%)	17 (1.9%)
High	708 (56.9%)	535 (60%)
Normal	526 (42.2%)	339 (38%)
HDL	1.53 ± 0.01	1.50 ± 0.01
Uric acid	348.89 ± 2.44	359.2 ± 2.99

BMI: body mass index; WC: waist circumference; WHtR: waist-height ratio; SD: standard deviation; TSH: thyroid stimulating hormone; LDL: low-density lipoprotein; HDL: high-density lipoprotein. “^*∗*^” and bold text represent statistical significance.

**Table 2:** Gender differences in the prevalence of different thyroid diseases.

Item	Prevalence		Total	*p*
	Male	Female		
Thyroid dysfunction				**0.004 ** ^*∗*^
Hypothyroidism	1 (0.3%)	26 (2.8%)	27 (2.2%)	
Subclinical hypothyroidism	34 (10.3%)	130 (14.2%)	164 (13.2%)	
Thyroid hyperfunction				
Hyperthyroidism	8 (2.4%)	22 (2.4%)	30 (2.4%)	
Subclinical hyperthyroidism	3 (0.9%)	21 (2.3%)	24 (1.9%)	
Thyroid cancer	3 (0.9%)	9 (1.0%)	12 (1.0%)	
Thyroma	1 (0.3%)	2 (0.2%)	3 (0.2%)	
Thyroid nodules	233 (70.8%)	538 (58.7%)	771 (61.9%)	
Thyroid antibody positive	46 (14.0%)	168 (18.3%)	214 (17.2%)	

“^*∗*^” and bold text represent statistical significance.

**Table 3:** Comparison of gender differences in the prevalence of thyroid diseases in different age groups.

Age (years)	Male			Female			Total			*χ * ^2^	*p*
	*N* of surveys	*N* of illnesses	Prevalence (%)	*N* of surveys	*N* of illnesses	Prevalence (%)	*N* of surveys	*N* of illnesses	Prevalence (%)		
18–29	72	24	33.33	176	83	47.16	248	107	43.15	3.98	**0.046 ** ^*∗*^
30–39	130	43	33.08	264	150	56.82	394	193	48.98	19.65	**<0.001 ** ^*∗*^
40–49	146	64	43.84	323	206	63.78	469	270	57.57	16.37	**<0.001 ** ^*∗*^
50–59	215	117	54.42	416	298	71.63	631	415	65.77	18.66	**<0.001 ** ^*∗*^
60–69	110	58	52.73	190	134	70.53	300	192	64.00	9.58	**0.002 ** ^*∗*^
≥70	36	23	63.89	58	45	77.59	94	68	72.34	2.08	0.149
Total	709	329	46.40	1427	916	64.19	2136	1245	58.29	61.64	**<0.001 ** ^*∗*^

*N*: number. “^*∗*^” and bold text represent statistical significance.

**Table 4:** Differences of thyroid prevalence under different obesity parameters.

Item	Total	BMI				Central obesity	
		Normal (18.5 ≤ BMI < 24)	Underweight (<18.5)	Overweight (24 ≤ BMI < 28)	Obesity (≥28)	Yes	No
Thyroid dysfunction							
Hypothyroidism	27	11 (40.7%)	1 (3.7%)	10 (37%)	5 (18.5%)	9 (33.3%)	18 (66.7%)
Subclinical hypothyroidism	164	82 (50.0%)	11 (6.7%)	52 (31.7%)	19 (11.6%)	44 (26.8%)	120 (73.2%)
Thyroid hyperfunction							
Hyperthyroidism	30	17 (56.7%)	5 (16.7%)	8 (26.7%)	0 (0%)	2 (6.7%)	28 (93.3%)
Subclinical hyperthyroidism	24	10 (41.7%)	2 (8.3%)	10 (41.7%)	2 (8.3%)	5 (20.8%)	19 (79.2%)
Thyroid cancer	12	3 (25.0%)	1 (8.3%)	5 (41.7%)	3 (25%)	5 (41.7%)	7 (58.3%)
Thyroid nodules	771	366 (47.5%)	28 (3.6%)	282 (36.6%)	95 (12.3%)	264 (34.2%)	507 (65.8%)
Positive thyroid antibody	214	124 (57.9%)	18 (8.4%)	58 (27.1%)	14 (6.5%)	44 (20.6%)	170 (79.4%)
Thyroma	3	3 (100.0%)	0 (0%)	0 (0%)	0 (0%)	0 (0%)	3 (100.0%)
Normal	891	469 (52.6%)	50 (5.6%)	289 (32.4%)	83 (9.3%)	218 (24.5%)	673 (75.5%)
*χ * ^2^	—	—	11.33	15.21	21.70	36.59	
*p*	—	—	0.184	0.055	**0.006 ** ^*∗*^	**<0.001 ** ^*∗*^	

BMI: body mass index; WC: waist circumference. Central obesity: male waist circumference ≥90 cm and female waist circumference ≥85 cm. “^*∗*^” and bold text represent statistical significance.

**Table 5:** The difference of thyroid disease between recessive and positive autoantibody groups.

Item	TPOAb		TgAb	
	+ (%) *n* = 418	−(%) *n* = 1716	+ (%) *n* = 313	−(%) *n* = 1812
Thyroid dysfunction				
Hypothyroidism	12 (2.9%)	22 (1.3%)	1 (0.3%)	26 (1.4%)
Subclinical hypothyroidism	31 (7.4%)	133 (7.8%)	26 (8.3%)	138 (7.6%)
Thyroid hyperfunction				
Hyperthyroidism	6 (1.4%)	24 (1.4%)	7 (2.2%)	23 (1.3%)
Subclinical hyperthyroidism	16 (3.8%)	20 (1.2%)	1 (0.3%)	23 (1.3%)
Thyroid cancer	2 (0.5%)	10 (0.6%)	0 (0%)	12 (0.7%)
Thyroid nodules	141 (33.7%)	628 (36.6%)	113 (36.1%)	656 (36.2%)
Positive thyroid antibody	46 (11%)	168 (9.8%)	45 (14.4%)	169 (9.3%)
Thyroma	1 (0.2%)	2 (0.1%)	0 (0%)	3 (0.2%)
Total	255 (61.0%)	1007 (58.7%)	193 (61.7%)	1050 (57.9%)
*p*	**<0.001 ** ^*∗*^		**<0.001 ** ^*∗*^	

TPOAb: thyroid peroxidase antibody; TgAb: thyroglobulin antibody; “+”: positive autoantibody; “−”: negative autoantibodies. “^*∗*^” and bold text represent statistical significance.

**Table 6:** Comparison of gender differences in autoantibody positive rates in different age groups.

Age (years)	TPOAb (+)		*p*	TgAb (+)		*p*
	Male	Female		Male	Female	
18–29	17 (11.7%)	38 (13.9%)	0.944	7 (7.1%)	28 (13.1%)	0.463
30–39	30 (20.7%)	51 (18.7%)		17 (17.2%)	42 (19.6%)	
40–49	31 (21.4%)	63 (23.1%)		22 (22.2%)	54 (25.2%)	
50–59	44 (30.3%)	74 (27.1%)		35 (35.4%)	60 (28.0%)	
60–69	16 (11.0%)	31 (11.4%)		14 (14.1%)	23 (10.7%)	
≥70	7 (4.8%)	16 (5.9%)		4 (4.0%)	7 (3.3%)	
Total	145 (34.7%)	273 (65.3%)		99 (31.6%)	214 (68.4%)	

TPOAb: thyroid peroxidase antibody; TgAb: thyroglobulin antibody; “+”: positive autoantibody; “−”: negative autoantibodies.

**Table 7:** Differences of four thyroid hormones in different genders.

Gender	UIC (ug/L)		*U*	*p*	TSH (mIU/L)		*U*	*p*	FT4 (pmol/L)		*U*	*p*	FT3 (pmol/L)		U	*p*
	Median	(P25, P75)			Median	(P25, P75)			Median	(P25, P75)			Median	(P25, P75)		
Male	159.32	(107.23, 221.92)	461226.5	**0.004 ** ^*∗*^	1.67	(1.21, 2.45)	459022.5	**0.001 ** ^*∗*^	17.14	(15.01, 19.50)	3428.5	**0.002 ** ^*∗*^	7.35	(5.67, 9.90)	239.0	0.444
Female	147.30	(89.09, 223.74)			1.88	(1.23, 2.90)			15.5	(13.48, 17.73)			6.15	(4.91, 8.75)		

UIC: urinary iodine concentration; TSH: thyroid stimulating hormone; FT4: free thyroxine; FT3: free triiodothyronine. “^*∗*^” and bold text represent statistical significance.

**Table 8:** The influence of different urine iodine concentration on the prevalence of thyroid diseases.

UIC (*μ*g/L)	*N* of survey	Thyroid cases		Prevalence (%)
		Yes	No	
0∼	156	101	55	64.7
50∼	294	203	91	69.0
100∼	292	181	111	62.0
150∼	275	161	114	58.5
200∼	279	142	137	50.9
250∼	158	71	87	44.9
300∼	165	91	74	55.2
350∼	129	64	65	49.6
400∼	166	83	83	50.0
450∼	132	78	54	59.1
500∼	90	70	20	77.8
Total	2136	1245	891	58.3

*N*: number; UIC: urinary iodine concentration.

**Table 9:** Multivariate logistic regression analysis of the prevalence of thyroid diseases.

Variables	*β*	S.E.	Wald	*p*	OR	95%CI
Gender						
Male (ref)						
Female	0.91	0.16	31.45	**<0.001 ** ^*∗*^	2.49	1.81–3.43
Age						
18–29 (ref)				<0.001		
30–39	1.65	0.47	12.28	0.213	1.44	2.07–13.06
40–49	1.13	0.43	6.80	**<0.001 ** ^*∗*^	2.44	1.32–7.22
50–59	0.89	0.43	4.34	**0.009 ** ^*∗*^	3.09	1.05–5.63
60–69	0.52	0.42	1.55	**0.037 ** ^*∗*^	5.20	0.74–3.85
≥70	0.44	0.44	0.99	0.319	1.55	0.66–3.66
BMI						
18.5 ≤ BMI < 2 (ref)				0.320		
BMI<18.5	−0.30	0.40	0.57	0.452	0.74	0.34–1.63
24 ≤ BMI<28	0.19	0.28	0.44	0.506	1.20	0.70–2.08
BIM ≥ 28	−0.04	0.28	0.02	0.876	0.96	0.55–1.66
Smoking						
Yes (ref)						
No	0.24	0.24	0.93	0.335	1.27	0.78–2.04
Region						
Suburb (ref)						
Urban	0.07	0.17	0.15	0.696	1.07	0.77–1.48
Season						
Spring (ref)			2.05	0.562		
Summer	−0.03	0.21	0.02	0.881	0.97	0.64–1.47
Autumn	−0.26	0.21	1.63	0.201	0.77	0.51–1.15
Winter	0.07	0.24	0.08	0.777	1.07	0.66–1.73
Family history of thyroid disease						
Yes (ref)						
No	−0.85	0.23	13.96	**<0.001 ** ^*∗*^	0.43	0.27–0.67
Hyperlipidemia						
Yes (ref)						
No	−0.15	0.20	0.56	0.456	0.86	0.58–1.28
Hyperuric acidemia						
Yes (ref)						
No	−0.01	0.17	0.00	0.968	0.99	0.71–1.39
Hypertension						
No (ref)						
Yes	0.15	0.18	0.68	0.409	1.16	0.82–1.65
Gout disease						
No (ref)						
Yes	0.13	0.23	0.33	0.567	1.14	0.72–1.81
TPOAb						
− (ref)						
+	0.43	0.22	3.74	0.053	1.54	0.99–2.38
TgAb						
− (ref)						
+	−0.36	0.25	2.15	0.143	0.70	0.43–1.13
Triglyceride						
Normal (ref)			2.38	0.304		
L	0.26	0.43	0.36	0.547	1.30	0.56–3.00
H	0.24	0.16	2.20	0.138	1.27	0.92–1.75
Total cholesterol						
Normal (ref)			0.15	0.926		
L	−0.09	1.07	0.01	0.934	0.92	0.11–7.41
H	−0.06	0.16	0.15	0.699	0.94	0.69–1.29
LDL						
Normal (ref)			0.91	0.635		
H	0.17	0.18	0.86	0.354	1.18	0.83–1.68
HDL						
Normal (ref)						
L	0.51	0.63	0.65	0.420	1.66	0.49–5.67
H	−0.08	0.16	0.21	0.647	0.93	0.67–1.28
Serum vitamin D3						
Inadequate						
Amplitude	0.01	0.18	0.01	0.939	1.01	0.71–1.44

TPOAb: thyroid peroxidase antibody; TgAb: thyroglobulin antibody; “+”: positive autoantibody; “−”: negative autoantibodies; LDL: low-density lipoprotein; HDL: high-density lipoprotein. “^*∗*^” and bold text represent statistical significance. “^*∗*^” and bold text represent statistical significance.

## Data Availability

The datasets used and analyzed in the current study are available from the corresponding author on reasonable request.
